# Pathological Clavicle Fracture: Initial Presentation of Intrahepatic Cholangiocarcinoma

**DOI:** 10.7759/cureus.7094

**Published:** 2020-02-24

**Authors:** Koray Başdelioğlu

**Affiliations:** 1 Orthopedics and Traumatology, Istanbul Oncology Hospital, Istanbul, TUR

**Keywords:** pathological fracture, clavicula, metastasis, liver, intrahepatic cholangiocarcinoma

## Abstract

Intrahepatic cholangiocarcinoma (ICC) is the second most common tumor of the liver and accounts for 3% of all gastrointestinal tumors. Bone metastasis due to ICC is extremely rare. In this case report, a patient with pathological clavicle fracture as the first presentation of ICC was reported. A lytic mass causing a fracture in the middle part of the left clavicle was detected in a 75-year-old female patient who had sudden and severe pain in her left shoulder while getting up from her seat. Blood tests were normal except gamma-glutamyl transferase 575 U/L (0-38 U/L), alkaline phosphatase 259 U/L (0-105 U/L), direct bilirubin 5.1 mg/dl (0-0.2 mg/dl) and carcinoembryonic antigen 5.1 ng/ml (0-5 ng/ml). Positron emission tomography (PET-CT) revealed a mass in the liver and metastasis to the proximal femur and peritonei carcinomatosis. Pathological clavicle fracture was treated surgically and liver biopsy was performed by an interventional radiologist in the same session. The pathology result was reported as ICC. During the follow-up of the patient, a pathological proximal femur fracture also occurred. This fracture was also treated with total tumor hip replacement. Metastasis of ICC to the bone is extremely rare and should be kept in mind in the differential diagnosis in patients presenting with pathological bone fracture and liver mass.

## Introduction

Intrahepatic cholangiocarcinoma (ICC) is a malignant tumor originating from small bile ducts in the liver [1]. ICC is the second most common tumor of the liver after hepatocellular carcinoma and accounts for 3% of all gastrointestinal cancer [2-3]. It is seen in the Western world at an average rate of one to two per 100,000 [4-6]. Biliary inflammation and fibrosis caused by primary sclerosing cholangitis and primary biliary cirrhosis, hepatolithiasis, hepatitis B infection, hepatitis C infection, parasitic infection, obesity, alcohol drinking, tobacco smoking, and host genetic polymorphisms are the important risk factors of ICC [7].

The tumor usually metastasizes to regional lymph nodes with lymphatic spread and to organs such as the adrenal gland, brain, lung, and peritoneum with hematogenous spread [1,8]. ICC metastasis to the appendicular skeleton is extremely rare. There are very few publications in the literature on this subject [9-12]. Three of these publications were related to metastasis of the cholangiocarcinoma to the humerus and one was related to metastasis to the distal fibula.

This case report is the first case of ICC presenting with a pathological clavicle fracture in the literature.

## Case presentation

**Figure 1:**
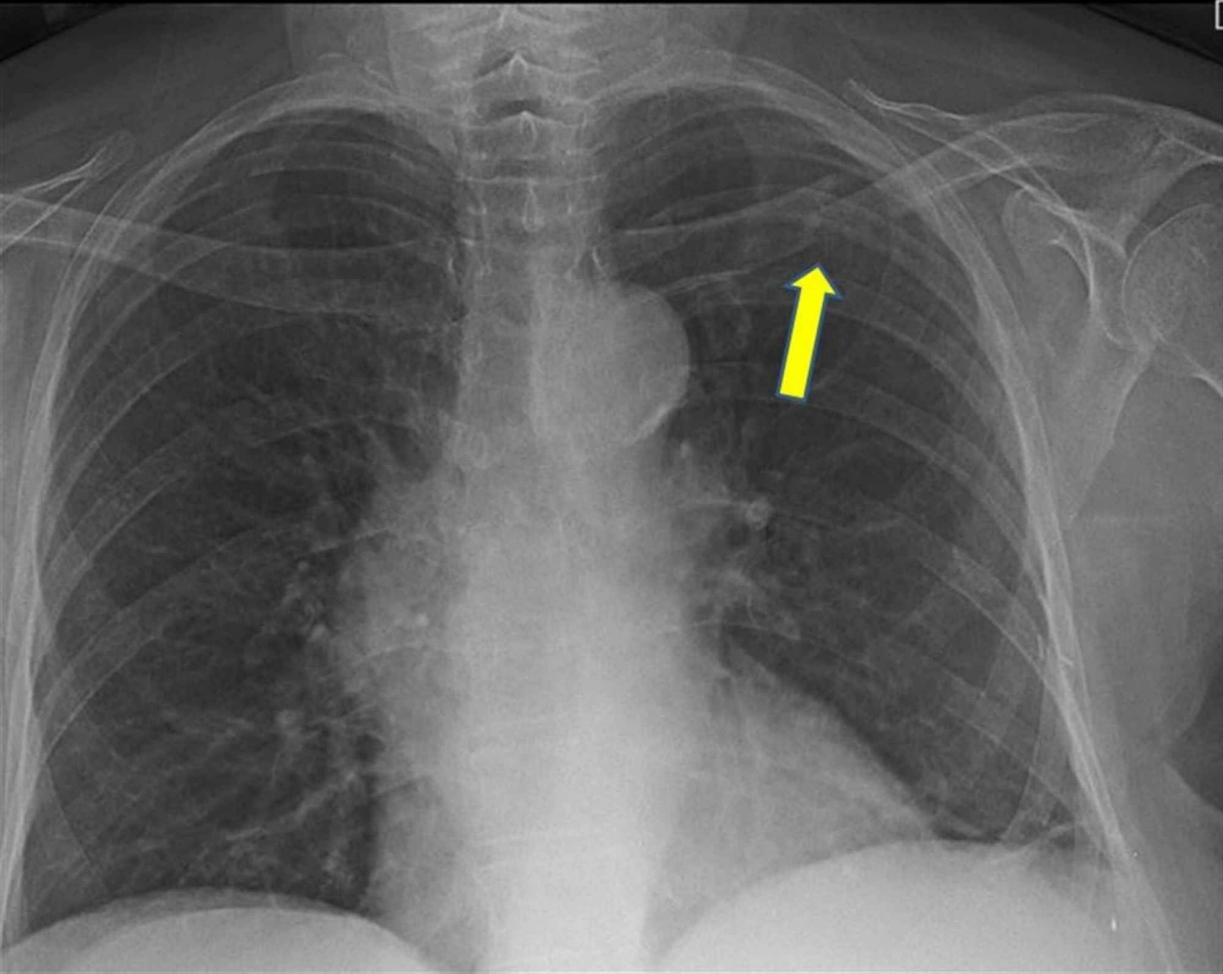
Fracture of the left clavicle in the X-ray

A 75-year-old female patient presented to our emergency department with left shoulder pain. The patient reported a sudden and severe pain in his left shoulder while getting up from the chair. On examination, the patient had severe tenderness, edema, and crepitation in the middle part of the left clavicle. The neurovascular examination was normal. The X-ray was performed first, and it showed lytic areas accompanying the fracture of the midshaft left clavicle (Figure 1).

**Figure 2:**
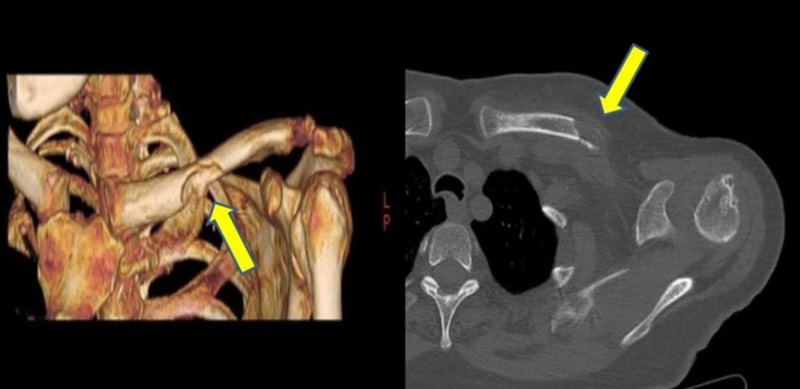
Lytic mass lesion in the middle of the left clavicula and pathologic fracture in the CT image

Computed tomography (CT) was performed, which showed a 25x18mm lytic mass lesion in the middle of the left clavicula and the pathologic fracture caused by this mass lesion. The anterior cortex was destructed by the mass (Figure 2).

**Figure 3:**
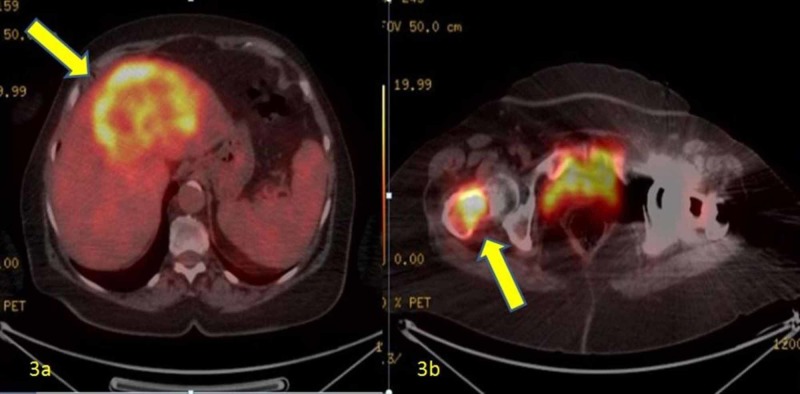
3a: Hypermetabolic malignant mass with axial dimensions of 12x10 cm in the liver in PET-CT; 3b: Metastatic mass in the proximal femur in PET-CT PET-CT: positron emission tomography-computed tomography

Laboratory tests revealed her hemoglobin 13.6 gr/dl (11-15 gr/dl), white blood cell 8,04 10 ^ 9 /L (4-10 10 ^ 9 /L), platelet 208 10 ^ 9 /L (130- 400 10 ^ 9 /L), creatinine 0.62 mg/dl (05-0.9 mg/dl), aspartate aminotransferase 37 U/L (0-40 U/L), alanine aminotrasferase 32 U/L (8-41 U/L), gamma glutamyl transferase 575 U/L (0-38 U/L), alkaline phosphatase 259 U/L (0-105 U/L), direct bilirubin 5.1 mg/dl (0-0.2 mg/dl) ), total bilirubin 0.98 mg/dl (0-1 mg/dl), alpha fetoprotein 5.9 ng/ml (0-10 ng/ml), carcinoembryonic antigen 5.1 ng/ml (0-5 ng/ml) ml). The patient underwent positron emission tomography (PET-CT), considering that the mass causing the calvicula fracture may be a metastatic focus. PET-CT revealed a central necrosis hypermetabolic malignant mass with axial dimensions of 12x10 cm in the liver (Figure 3a). Peritonei carcinomatosis and a metastatic mass in the proximal femur (Figure 3b) was also seen. The PET-CT was normal except for these findings.

**Figure 4:**
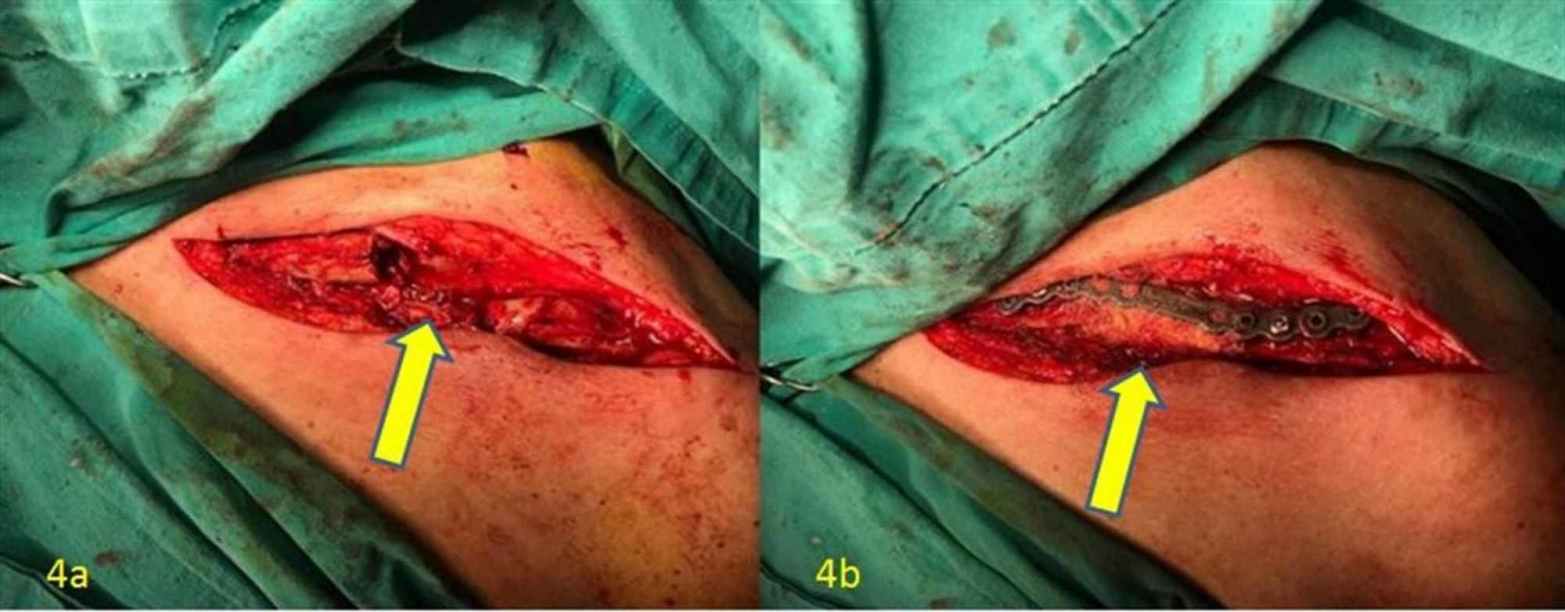
4a: Resection of the mass and the defect; 4b: Allograft reconstruction and osteosynthesis of the metastatic clavicle fracture

**Figure 5:**
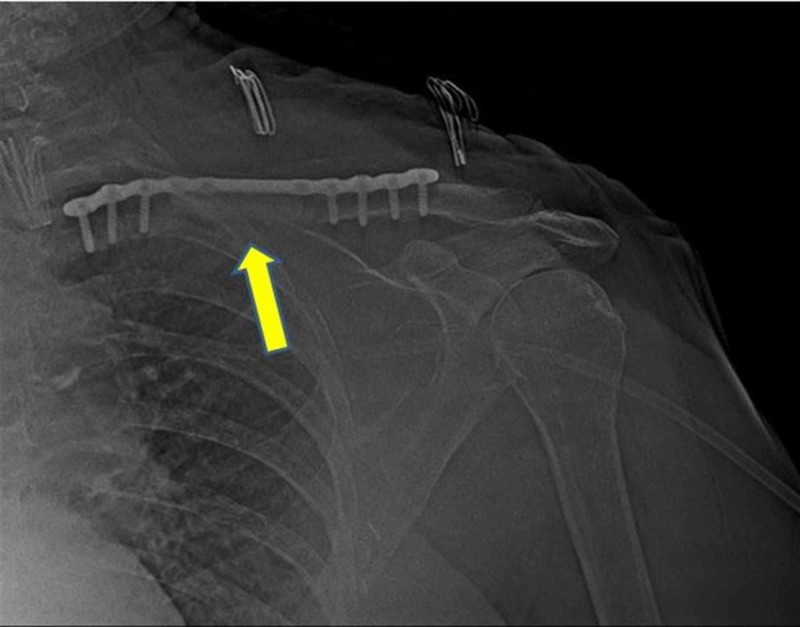
Postoperative X-ray of the left clavicle

Surgery was planned for the pathological clavicle fracture. In the same session, a liver biopsy was performed by the interventional radiologist. The clavicle segment containing the metastatic mass causing the fracture was excised medially and laterally leaving 1 cm of clear surgical margin (Figure 4a). After excision, the defective area was reconstructed with a strut allograft and spongious allograft. The fixation was achieved with an anatomic clavicle plate (Figure 4b). Postoperative X-ray was performed (Figure 5). No complications were encountered intraoperatively and postoperatively. However, the patient was admitted to the emergency department of our hospital with severe right hip pain in the second postoperative week. Total hip tumor prosthesis was performed with the diagnosis of a pathological proximal femur fracture in the patient with a known metastatic mass in the right femoral head and neck.

Pathology was reported as carcinoma metastasis and ICC. Immunohistochemical results were cytokeratin 7 positive, cytokeratin 20 weak positive, thyroid transcription factor 1, and GATA-3 negative. Following the diagnosis of ICC, chemotherapy and radiotherapy treatments were recommended by the medical oncology and radiation oncology departments. The patient refused these treatments.

## Discussion

ICC is a rare tumor and results in early mortality. Five-year survival in patients undergoing surgical resection is less than 5% [13-14]. Metastasis in the ICC is mostly lymphatic to the regional lymph nodes and hematogenously to the organs such as the lungs, adrenal gland, brain, and peritoneum [1,8]. Bone, and especially the appendicular skeletal metastasis due to ICC, is extremely rare. In this case report, a patient with a pathological clavicle fracture as the first presentation of ICC was reported.

There are very few publications in the literature reporting bone metastasis secondary to ICC [9-12,15]. In the literature, Carlisle et al., Lahrach et al., and Federico et al. reported metastatic humeral fractures related to ICC [9-11]. Karanjia et al. reported a case with distal fibular metastasis secondary to ICC [12]. MacKenzie et al. treated distal femoral metastasis with en bloc resection and tumor prosthesis in a patient with known ICC [14]. Chindaprasirt et al. presented an ICC case with scapular bone and surrounding soft metastasis [15]. As can be seen, there are very few publications in the literature reporting cases of bone metastasis due to ICC. The case I presented is the only case in the literature that was diagnosed with ICC as a result of pathological clavicle fracture and underwent surgical treatment for both pathological clavicle fracture and pathological proximal femoral fracture.

There are publications in the literature reporting on metastatic clavicle fractures due to hepatocellular carcinoma, renal cell carcinoma, and prostate, thyroid and colon tumors [16]. However, there are no reports of pathological clavicle and proximal femoral fractures secondary to ICC.

The main goal of treatment in such cases is to eliminate pain, allowing early mobility and mobilization to optimize functional capacity. Thus, it is aimed to increase the quality of life.

## Conclusions

Metastasis of ICC to bone is extremely rare. However, the first presentation may be with fracture or bone pain. ICC, as a differential diagnosis, should always be kept in mind in patients who have a pathological bone fracture due to minor trauma or bone pain with liver mass.
